# Transient high glycaemic intake in the last trimester of pregnancy increases offspring birthweight and postnatal growth rate in sheep: a randomised control trial

**DOI:** 10.1111/j.1471-0528.2009.02149.x

**Published:** 2009-04-08

**Authors:** NA Smith, FM McAuliffe, K Quinn, P Lonergan, ACO Evans

**Affiliations:** a School of Agriculture Food Science and Veterinary Medicine, University College DublinBelfield, Dublin, Ireland; b UCD Obstetrics and Gynaecology, School of Medicine and Medical Science, National Maternity HospitalDublin, Ireland; c UCD Conway Institute of Biomolecular and Biomedical Research, College of Life Sciences, University College DublinBelfield, Dublin, Ireland

**Keywords:** Birthweight, fetal programming, maternal nutrition

## Abstract

**Objective:**

Investigate the effect of transient hyperglycemic intake (analogous to snacking on high glycaemic foods) in the third trimester of pregnancy on offspring birthweight and subsequent growth in sheep.

**Design:**

Randomised trial.

**Setting:**

University research farm.

**Sample:**

Third trimester pregnant ewes.

**Methods:**

Ewes were blocked on weight, age and litter size and were randomly assigned to receive oral administration of 100 ml of propylene glycol (PG; *n* = 51) or 100 ml of water (control, C; *n* = 53) twice/day. Twice during treatment, 12 ewes from each group were selected and blood samples collected to determine the glucose and insulin response to treatment.

**Main outcome measures:**

At birth, blood was collected from the lambs, their body dimensions measured and body weights recorded at 0, 6 and 12 weeks of age after which lambs were slaughtered when they reached 40 kg live weight.

**Results:**

Administration of PG elevated (*P* < 0.05) plasma glucose and insulin concentrations for 2 hours post administration compared with control ewes. Lambs (C: *n* = 80; PG: *n* = 70) born to ewes fed high glycaemic meals had higher birthweights (C: 5.01 ± 0.18 kg; PG: 5.27 ± 0.22 kg, *P* = 0.032), plasma glucose concentrations (*P* = 0.001) and ponderal index (weight/height^3^, *P* = 0.043) and reached a similar (*P* > 0.05) slaughter carcass weight (C: 20.0 ± 0.51 kg; PG: 20.6 ± 0.55 kg) at an earlier age (PG: 166.0 ± 13.2; C: 183.4 ± 13.8 days, *P* = 0.039) compared with control lambs.

**Conclusions:**

Transient high glycaemic intakes in the third trimester of pregnancy resulted in heavier offspring at birth that had faster growth rates in early postnatal life. This animal model is relevant for studying the relationship between maternal diet, fetal size and the risk of childhood obesity.

## Introduction

Experimental evidence in humans has established that maternal weight and maternal weight gain during pregnancy significantly influences infant birthweight in humans.^1^–^4^ Fetal macrosomia (large for dates with birthweight >4.0 Kg) is associated with an increased risk of maternal perineal trauma^5^ and an increased risk of trauma to the infant.^6^,^7^ Additionally, recent studies have found that infants born at the highest end of the distribution for weight were at a higher risk of being obese in childhood, adolescence and adulthood when compared with normal sized infants.^8^,^9^ In humans, the source of maternal glucose originates either from the maternal liver or from the maternal diet. High maternal glucose concentrations are thought to increase maternal weight gain, result in feto-placental overgrowth as well as a higher risk of fetal macrosomia, while low maternal glycaemic diets result in normal maternal weight gain and produces infants with birthweights between the 25th and 50th percentile.^10^ A recent study has shown a relationship between elevated maternal glucose concentrations during gestation (below those levels diagnostic of diabetes) and increased birthweight.^11^ With the above evidence in mind, it is apparent that there is a need to control the level of glucose consumption during pregnancy to help reduce maternal and fetal trauma at parturition, as well as reducing the risk of obesity related adult diseases later in life.

Consumption of high glycaemic index diets increase postprandial glucose peaks as well as increasing fasting glucose levels compared to low glycaemic diets.^12^ In addition, lifestyle choices mean that pregnant women in the developed world often consume high glycaemic snacks between meals that may temporarily increase circulating glucose and insulin concentrations.^13^ The impact high glycaemic maternal diet, causing transient elevations in glucose concentrations on fetal development has not been studied.

Glucose is the main energy substrate for fetal growth^14^–^16^ and fetal insulin permits greater glucose and amino acid entry into cells, increasing metabolism and, ultimately, fetal growth.^17^ Propylene glycol is a 3-carbon compound (C_3_H_8_O_2_) derived from propylene. In ruminants, it increases ruminal propionate, which is then transformed to pyruvate and eventually converted to glucose in the liver via oxaloacetate^18^,^19^ Propylene glycol is widely used in ruminants as an oral drench and is a gluconeogenic precursor that elicits a glucose and insulin response.^18^,^19^

The aim of this study was to investigate the consequences of transient, intermittent high glycaemic intake during the last trimester of pregnancy on neonatal size and postnatal growth in an animal model. To achieve this aim, we studied lambs born to pregnant ewes that received a twice daily high glycaemic oral dose of propylene glycol or water (control) in addition to their normal meals during the last trimester of pregnancy.

## Materials and methods

This randomised controlled trial was approved by the UCD Animal Research Ethics Committee. All experimental procedures involving animals were licensed by the Department of Health and Children (a Department of the Irish Government), in accordance with the Cruelty to Animals Act (Ireland 1897) and European Community Directive 86/609/EC.

Sheep are considered a useful model for humans in which to investigate the physiology of pregnancy and fetal development^20^ as they have a body weight of 65 to 85 kg, have a 17 day (average) reproductive cycle, usually have one or two lambs per pregnancy, have a relatively long gestation of 147 days (range 142–152 days), are amenable to reproduction, nutrition and surgical manipulation (as required) and can tolerate multiple observations (e.g. ultrasound) and tissue collections (e.g. blood sampling).

### Experiment 1: Establishment of propylene glycol dosage

A preliminary experiment was carried out on nine non pregnant 4-year-old ewes to investigate the effects of different doses of propylene glycol on their glucose and insulin response. Ewes were randomly assigned to one of three treatment groups and were administered orally either; (i) 50 ml water (*n* = 3), (ii) 50 ml propylene glycol (*n* = 3), (iii) 100 ml propylene glycol (*n* = 3). Blood samples (4 ml collected into a heparinised and a sodium oxalate vacutainer [Unitech, Dublin, Ireland]) were taken at −30, 0, 30, 60, 90, 120, 150, 180, 240 and 300 minutes relative to dosing with water or propylene glycol. All blood was centrifuged at 1600 × g for 20 minutes at 4ºC. The plasma was separated and stored at −20ºC until analysis.

### Experiment 2: Effect of feeding high glycaemic meals to ewes during the last trimester of pregnancy on lamb weight and growth rates

**Figure 1:**
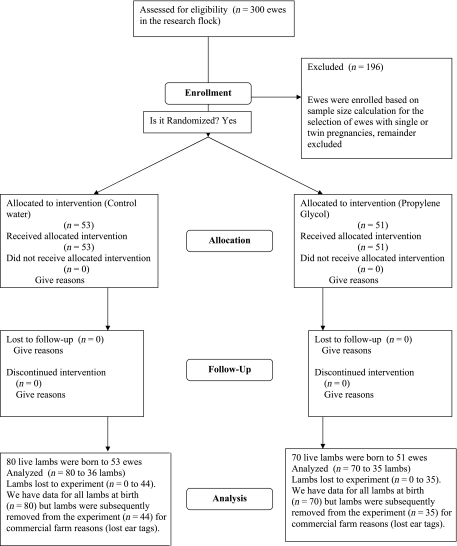
CONSORT flowchart.

A schematic presentation of the animals and treatments is presented in the consort flowchart (Figure 1). To synchronise mating and subsequent lambing, an intravaginal progestagen pessary (30 mg flugestone acetate; Chronogest, Intervet, Boxmeer, the Netherlands) was inserted into approximately 300 Suffolk-cross ewes ranging in age from 2 to 5 years for 12 days, which were later mated with rams at a ratio of one ram per seven ewes. To determine litter size and confirm stage of gestation, ewes were scanned using transabdominal ultrasonography 75 days after ram introduction and remained outdoors on permanent grassland until they were housed at 82 days gestation. From housing until parturition, ewes were offered grass silage *ad libitum*. Based on established criteria,^21^ the energy requirements of the ewes was estimated to be 12 MJ at 90 days gestation rising to 20 MJ just before lambing. This was provided by offering grass silage *ad libitum* and supplemented with 500 g to 700 g of concentrate meal.

On day 98 of gestation (the first day of the third trimester), 104 ewes were chosen from the main flock after a sample size calculation that indicated a need for at least 50 animals in each of the two groups (see below). To do this, ewes from the main flock were excluded if they were not pregnant or were carrying three or more fetuses. Remaining animals were then divided into large holding pens containing ewes with single or twin fetuses. They were then randomly presented by the stock person to be weighed and their ear tag number (assigned at birth by the Department of Agriculture) recorded until we had assembled 50 ewes with single fetuses and 54 ewes with twin fetuses. Upon completion of weighing, ewes were alternately assigned to one of the two treatment groups (see below) from the list of ear tag numbers (recorded in random order during weighing). The two treatment groups were 100 ml twice per day (0800 and 1600) of water (*n* = 53) or propylene glycol (*n* = 51) (Inform Nutrition Ireland Ltd, Cork, Ireland) from the afternoon of day 98 of gestation to the first signs of parturition about day 147. The propylene glycol (200 ml per day) provided 4.4 MJ per day of energy to the propylene glycol treated ewes.

On day 109 of gestation, six ewes from each of the treatment groups (PG or C treated single and twin bearing ewes) were randomly selected. The same animals were selected again on day 140 of gestation. On each day, blood samples (4 ml collected into a heparinised and a sodium oxalate vacutainer tubes (Unitech, Dublin, Ireland) were taken every 30 to 60 minutes from 90 minutes before the first treatment of propylene glycol or water. All blood was centrifuged at 1600 × g for 20 minutes at 4ºC. The plasma was separated and stored at −20ºC until analysis. We chose to characterise the glucose and insulin response to treatment on day 109 of gestation as this was 10 days after the start of treatment and on day 140 of gestation as this was the latest day during treatment that we could predict no ewes would have started lambing (labour). Six ewes per groups were chosen based on previous experience of the variation among animals (contributing to a sample size calculation) for concentrations of glucose and insulin.

At birth, lambs were given individually identifying punch earl tags, lambs were weighed, their gender was recorded, blood samples were taken before their first feed and were analysed for plasma glucose and IGF-I concentrations. Body dimensions (head circumference, height, thoracic circumference, jaw circumference, crown rump length, body length, inside limb length) were measured within 24 hours of birth using a standard measuring tape. Rohrer’s ponderal index in newborns (birthweight/height × 100)^3^ has been used as an indicator of fetal growth status, especially to assess asymmetrical intrauterine growth retardation. A ponderal index was calculated as weight/height^3^ expressed as kg/cm^3^. In humans, the ponderal index incorporates standing height, i.e. the length of the spine and long bones, whereas in quadrupeds, the length of the spine is not included in height.^22^ At approximately 1 day of age, ewes with their lambs were moved to pasture where they had *ad libitum* access to grass and water for the remainder of the experiment. Ewes nursing their lambs in all groups were maintained together on the same pasture and maintained equally at all times. The offspring were weighed at 6 and 12 weeks of age until they reached an approximate weight of 40 kg when they were slaughtered in a commercial abattoir house. Carcass weight and classification details (based on EUROP system) of each lamb was recorded. Arterial blood pressure of the lambs was measured using a noninvasive blood pressure monitor (CARDELL Veterinary Monitor 9401BP; SHARN Veterinary Inc. Tampa, FL, USA). At birth, a 2.0-cm-wide cuff with a circumference of 3 to 6 cm was placed on the metacarpus of the left forelimb where blood pressure could be measured from the dorsal metacarpal artery. This method has been validated for a number of species including sheep,^23^ dogs^24^ and pigs.^25^ To ensure an accurate reading, the cuff was selected so that its width was 40% of the limb diameter. Systolic, diastolic and mean arterial blood pressure and heart rate were recorded five times in each lamb. The highest and the lowest readings were discarded and an average was calculated from the three remaining readings. At all stages of data collection from the lambs, the treatments that their mothers received in the last trimester of gestation was unknown (data collection was blinded). Data were collected from all live lambs at birth (0 singles were born dead and in eight cases one twin of the pair was born dead; 3 C and 5 PG), but in cases where lambs subsequently lost their ear tags (and hence their identity), further data on them were not collected.

### Hormone assays

Plasma insulin concentrations were determined using a two-site fluoroimmunometric assay (Auto-DELFIA insulin; Perkin Elmer Life Sciences, Wallac Oy, Turku, Finland; catalogue no. B080-101) validated for bovine plasma (Ting *et al.* 2004). The intra-assay and inter-assay coefficients of variation for samples containing low (1.96 ± 0.12 μU/ml), medium (4.19 ± 0.49 μU/ml) and high (11.65 ± 2.12 μU/ml) insulin concentrations were all <6.5%. The analytical sensitivity of the assays is typically better than 0.5 μU/ml.

Plasma glucose concentrations were determined by enzymatic analysis using hexokinase and glucose-6-phosphate dehydrogenase enzymes to measure the formation of NADH photometrically (Randox imola system; Randox Laboratories Ltd., Co. Antrim, UK). The mean inter-assay coefficients of variation for samples containing low (6.15 ± 0.04 mmol/l) and high (16.27 ± 0.14 mmol/l) glucose concentrations were 2.82 and 2.05% respectively. The sensitivity of the assay was 0.64 mmol/l.

Plasma IGF-I concentrations were measured by double-antibody RIA (Armstrong *et al.* 1990). The samples were analysed as duplicate 100 μl aliquots and the sensitivity of the assay was 39 pg/ml. The mean inter-assay coefficients of variation for samples containing low (106.26 ± 9.04 pg/ml) medium (161.74 ± 9.04 pg/ml) and high (452.40 ± 64.43 pg/ml) IGF-I concentrations were 5.12, 13.56 and 10.58% respectively.

### Statistical analysis

All data were organised on several spread sheets by NS. These were circulated to all authors. KQ did all statistical analyses using SAS and circulated the output files. NS summarised the data and statistical findings which were then discussed and interpreted by all authors. All data are presented as means ± 95% confidence intervals, except for the Figures that are presented as the mean ± SEM (where the 95% confidence interval = 1.96 × SEM) and significance was accepted when *P* *<* 0.05. Area under the curve (AUC) was determined for glucose and insulin concentrations on day 109 and day 140 using the trapezoidal rule. This is the hormone response to treatment (increase) above the base line concentrations.^26^ Statistical comparisons between AUC for propylene glycol and water groups were completed using nonparametric analysis of variance. Characteristics of lambs at birth were analysed using factorial Analysis of variance (ANOVA) using PROC GLM with the model including the main effects of treatment, gender and litter size and interactions. Nonsignificant terms in the terms were excluded from final analyses. *Post hoc* analysis was performed with a Tukey’s HSD test as needed.

## Results

### Experiment 1: Propylene glycol dose

Insulin concentrations increased and returned to pre treatment concentrations by 150, 240 and 240 minutes in the three ewes that received 50 mls of propylene glycol (data not shown). Insulin concentrations increased in all three ewes that received 100 mls of propylene glycol and decreased to pre treatment values by 300 mins in one ewe and had not returned to pre treatment concentrations by 300 mins when blood sampling ended in the remaining two ewes (data not shown). Based on these findings, a dose of 100 ml was chosen for Experiment 2.

### Experiment 2: Effect of feeding high glycaemic meals to ewes during the last trimester of pregnancy on lamb weight and growth rates

**Figure 2:**
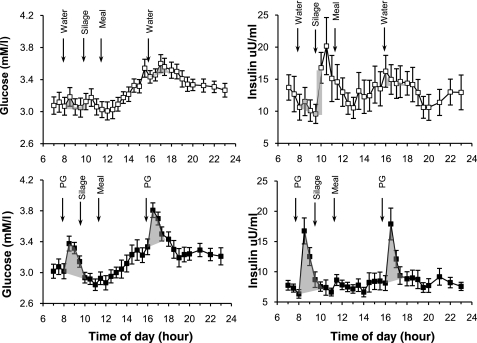
Glucose and insulin concentrations (mean ± SEM) on day 109 of gestation in ewes administered propylene glycol (*n* = 12) or water (*n* = 12) daily from day 98 of gestation. Ewes received 100 mls of propylene glycol (PG) or water (control) at 8.00 and again at 16:00 hours. Grass silage was fed *ad libitum* and replenished as shown and a concentrate meal was fed as shown. The glucose and insulin response to propylene glycol or water was calculated as area under the curve for the 2 hours post administration (shaded areas).

On day 98 of gestation (start of treatment), there were no differences among groups for the body weights of the ewes (81.0 kg ± 1.88, *n* = 104; *P* > 0.05). At both day 109 and day 140 of gestation, there was no difference (*P* > 0.30) in glucose or insulin concentrations between ewes with single or twin lambs (or interactions of litter size and treatment, *P* > 0.20); hence, the data were combined and are presented as propylene glycol (*n* = 12 ewes) versus water (*n* = 12 ewes) treatments at 109 and 140 days of gestation. Before treatment on day 109 of gestation, plasma glucose and insulin area under the curve (AUC) concentrations were not different (*P* > 0.05) between groups (Figure 2) and over the course of the entire 16 hour blood collection period, neither glucose nor insulin AUC concentrations were different among groups (*P* > 0.05). However, following treatment at 0800 hours on day 109 of gestation glucose (PG: 21.8 ± 6.9 mM/l; C: 6.1 ± 4.9 mM/l, *P* = 0.002) and insulin (PG: 504.8 ± 246.9 μU/ml; C: 1.3 ± 49.8 μU/ml, *P* = 0.006) 2 hour AUC were significantly greater in propylene glycol treated than water treated ewes. Similarly, at 16.00 hours, administration of propylene glycol resulted in a significantly larger glucose (PG: 38.8 ± 11.2 mM/l; C: 14.8 ± 10.6 mM/l, *P* = 0.006) and insulin (PG: 452.2 ± 143.7 μU/ml;C: 136.7 ± 114.4 μU/ml, *P* = 0.006) AUC response compared to water treated ewes (Figure 2).

**Figure 3:**
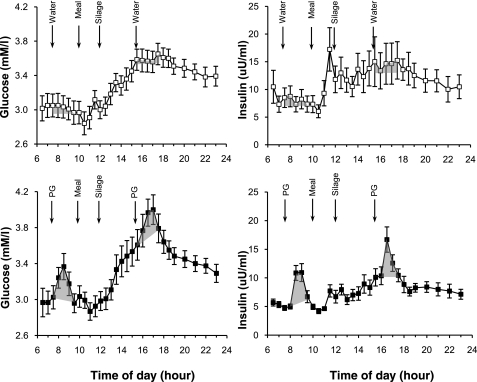
Glucose and insulin concentrations (mean ± SEM) on day 140 of gestation in ewes administered propylene glycol (*n* = 12) or water (*n* = 12) daily from day 98 of gestation. Ewes received 100 mls of propylene glycol (PG) or water (control) at 7.30 and again at 15.30 hours. Grass silage was fed *ad libitum* and replenished as shown and a concentrate meal was fed as shown. The glucose and insulin response to propylene glycol or water was calculated as area under the curve for the 2 hours post administration (shaded areas).

Before treatment on day 140 of gestation, plasma glucose and insulin AUC were not different among groups (Figure 3) and, as for day 109 of gestation, over the course of the whole 16 hour blood collection period, neither glucose nor insulin AUC concentrations were different among groups (*P* > 0.05). However, following administration of propylene glycol at 08.00 hours glucose (PG: 26.7 ± 10.8 mM/l; C: 2.21 ± 2.37 mM/l, *P* = 0.006) and insulin (PG: 339.6 ± 161.5 μU/ml; C: 28.6 ± 44.3 μU/ml, *P* = 0.002) AUC were significantly elevated during the 2 hours following administration compared with the water treated control group. At 16.00 hours, glucose AUC response to propylene glycol was not significantly larger (PG: 40.2 ± 20.1 mM/l; C: 14.8 ± 8.3 mM/l, *P* = 0.0733) however, insulin AUC were significantly elevated (PG: 157.9 ± 73.4 μU/ml; C: 53.1 ± 61.8 μU/ml, *P* < 0.0462) in the propylene glycol compared to the water treated control group (Figure 3).

### Characteristics of lambs at birth

The weight of lambs at birth was significantly affected by treatment, with lambs born to mothers fed propylene glycol being heavier (*P* < 0.05) than those born to control ewes given water (Table 1). Also, as expected, single lambs were heavier than twin lambs and male lambs were heavier than female lambs (data not shown). Lambs born to ewes treated with propylene glycol had higher (*P* < 0.001) glucose concentrations compared with lambs born to ewes from the control group, but fetal IGF-I concentrations were not different between groups (Table 1). There was no difference (*P* > 0.05) between the treatment groups for any of the body dimension measurements, but the ponderal index was significantly affected by treatment and gender, and there was a treatment-by-gender interaction with male lambs from propylene glycol treated ewes having a higher ponderal index than male lambs from control ewes (Table 1). Treatment did not significantly affect systolic (PG: 115.5 ± 9.9 mmHg; C 109.3 ± 7.8 mmHg), diastolic (PG: 84.0 ± 10.3 mmHg; C 77.1 ± 8.1 mmHg), mean (PG: 98.6 ± 10.6 mmHg; C 89.5 ± 7.6 mmHg) blood pressure or heart rate (PG: 165.5 ± 13.4 beats/min; C 164.4 ± 12.0 beats/min, *P* > 0.05) at birth.

**Table 1:** Characteristics (mean ± 95% confidence interval) of lambs born to ewes fed 100 ml propylene glycol (PG) or 100 ml water (control) twice per day from day 98 of gestation to term (about day 147)

Variables	Propylene glycol	Water	*P*-value
Gestation length (days)	147.9 ± 0.39 (*n* = 51)	147.6 ± 0.47 (*n* = 53)	T: 0.1917
Birthweight (kg)	5.27 ± 0.22 (*n* = 70)	5.01 ± 0.02 (*n* = 80)	T: 0.0324
Birth glucose (mM/l)	3.88 ± 0.57 (*n* = 61)	2.87 ± 0.33 (*n* = 76)	T: 0.001
Birth IGF-I (pg/ml)	629.8 ± 240.3 (*n* = 70)	774.8 ± 337.9 (*n* = 80)	T: 0.253
Height (cm)	36.66 ± 0.61 (*n* = 58)	36.85 ± 0.63 (*n* = 78)	T: 0.7628
Thoracic circumference (cm)	39.7 ± 0.8 (*n* = 58)	39.0 ± 0.8 (*n* = 78)	T: 0.1145
Ponderal index*			T: 0.0427
Male	119.8 ± 8.4 (*n* = 28)**	97.4 ± 5.5 (*n* = 45)***	G: 0.0372
Female	97.2 ± 7.1 (*n* = 30)***	105.2 ± 7.1 (*n* = 33)***	T × G: 0.0001
Growth rate 0 to 6 weeks (kg/day)	0.36 ± 0.18 (*n* = 55)	0.33 ± 0.02 (*n* = 55)	T: 0.0358
Growth rate 0 to12 weeks (kg/day)	0.31 ± 0.02 (*n* = 51)	0.29 ± 0.02 (*n* = 60)	T: 0.0022
Age at slaughter (days)	166.0 ± 13.2 (*n* = 35)	183.4 ± 13.8 (*n* = 36)	T: 0.0394
Carcass weight at slaughter (kg)	20.6 ± 0.55 (*n* = 35)	20.0 ± 0.51 (*n* = 36)	T: 0.1478

Probabilities are given for the effect of Treatment (T) or Gender (G) or their interaction (T × G). Where no probabilities are given for G or T × G they were not significant (*P* > 0.05). However, there was an effect (*P* < 0.001) of G and litter size (singleton versus twin lambs) on birthweight but there was no interactions (*P* > 0.05).

*Ponderal index = (birthweight/heights × 100)^3^.

**,***Values within variables with no common superscripts differ (*P* < 0.05).

Lambs born to mothers treated with propylene glycol had faster (*P* = 0.03) growth rates up to 6 weeks of age compared to lambs from water treated ewes (Table 1). Lambs from both groups were slaughtered at a similar (*P* > 0.05) weight (carcass weights were similar; Table 1); however, the offspring of ewes fed high glycaemic meals (propylene glycol) reached this weight at a younger age (*P* = 0.04) compared with control lambs (Table 1). There were no significant differences found for carcass weight (PG: 20.59 ± 0.55 kg; C: 20.02 ± 0.51 kg, *P* > 0.5) or conformation (*P* > 0.50) at slaughter between the two groups.

## Discussion

This study has found that high glycaemic intakes (analogous to snacking on high glycaemic foods) in the last trimester of pregnancy in ewes, giving transient elevations in glucose concentrations resulted in increased birthweight, basal plasma glucose concentrations and faster postnatal growth rates of their offspring.

It has been hypothesised that in humans high maternal blood glucose concentrations, as a result of diabetes, leads to an increase in nutrient transfer to the fetus.^27^ Fetal hyperglycaemia, then increases fetal pancreatic insulin secretion and subsequent fetal growth^28^, which can lead to complications in pregnancy.^29^ We have recently found that among women with gestational diabetes, commencement of the diabetic diet early in pregnancy which is isoenergetic with low glycaemic index foods for the carbohydrate component, was associated with less frequent macrosomia, when compared with late commencement.^30^ There is also an association between maternal glucose and fetal growth in women who do not have diabetes^11^,^28^ and eating primarily high glycaemic carbohydrates results in feto-placental overgrowth,^10^,^31^. In this study, we have now demonstrated that short duration, high glycaemic intake during late gestation that causes transient elevations in glucose and insulin concentrations, compared to the control ewes, had substantial effects to increase offspring birthweight and postnatal growth rates.

Women who were randomised to the high glycaemic diet delivered larger infants that were both heavier and larger in size.^31^ Our study is in agreement with this finding, as lambs born to ewes on a high gycaemic intake delivered heavier lambs with a higher ponderal index than control lambs (Table 1). This suggests that our animal model has relevance for investigation of fetal overgrowth mechanisms in humans.

We found that offspring born to ewes treated with propylene glycol in the last trimester of pregnancy had higher basal plasma glucose concentrations at birth, which could indicate impairment of the carbohydrate metabolism. These findings are in agreement with clinical investigations, which found that offspring born to mothers predisposed to gestational diabetes had elevated frequencies of impaired glucose tolerance.^32^ Additionally, offspring of rats displayed long-term alterations of glucose tolerance because of the induction of maternal gestational hyperglycaemia, which can lead to fetal and neonatal hyperinsulinism.^33^,^34^ An additional issue is that large for gestational age offspring, of diabetic mothers, are at significant risk of developing metabolic syndrome, which predisposes individuals to diabetes and cardivascular disease in childhood^35^ and adulthood.^36^ One of the criteria established for metabolic syndrome in this study was hypertension. In our study, we measured blood pressure in all lambs at birth, but found no significant differences (*P* > 0.05) between the two groups. Maternal undernutrition has been found to be associated with increased neonatal blood pressure in some studies,^37^,^38^ although others have found no such effect.^39^,^40^ However, it is possible that differences in blood pressure may not become apparent until later in life. We found that the high glycaemic intake in the last trimester of pregnancy resulted in offspring with faster rates of growth up until 12 weeks of age and reached their slaughter weight of about 40 kg earlier than the control lambs. We hypothesise that altered programming of fetal carbohydrate metabolism may also occur resulting in a persistence of the accelerated growth postnatally, even though the stimulus of maternal hyperglycaemia has been removed.

The limitations of this study are that we measured blood pressure in neonates and not in later life, it is possible that blood pressure changes may become evident in adolescence or adulthood (as discussed above). While we did not closely monitor the postnatal nutrition of the lambs, we feel that their diets were standardised as they were kept together in the same field with equal access to the dame diet. The strengths of this study are that it is a well-controlled randomised experiment with very good numbers (104 mothers and 150 offspring). Such a study would ethically and practically be difficult to perform on human pregnancy.

To our knowledge, there have been no previous animal studies carried out to investigate the effect of short duration high glycaemic intakes in the third trimester of pregnancy and its effects on offspring birthweight and growth rates. Our study shows for the first time, in a sheep model, that this resulted in an increased birthweight, basal plasma glucose concentrations and a faster postnatal growth rate in the offspring. Our hypothesis is that maternal hyperglycaemia stimulates fetal insulin production, which has a positive effect on fetal growth. There is evidence that maternal hyperglycaemia can result in chronic fetal hyperglycaemia and hyperinsulinaemia, which increases fetal fat mass and leptin synthesis within the fetal fat deposits.^13^

We conclude that our animal model has relevance for the investigation of the effects of transient maternal hyperglycaemia on offspring development and health. Altering the source and pattern of intake of maternal dietary carbohydrate may prove valuable in the management of pregnancies at risk for fetal overgrowth, trauma at parturition and possibly in the subsequent control of childhood obesity.
